# A TOTP-Based Enhanced Route Optimization Procedure for Mobile IPv6 to Reduce Handover Delay and Signalling Overhead

**DOI:** 10.1155/2014/506028

**Published:** 2014-02-09

**Authors:** Peer Azmat Shah, Halabi B. Hasbullah, Ibrahim A. Lawal, Abubakar Aminu Mu'azu, Low Tang Jung

**Affiliations:** ^1^Department of Computer & Information Sciences (CIS), Universiti Teknologi PETRONAS, 31750 Tronoh, Perak, Malaysia; ^2^Department of Computer Science, COMSATS Institute of Information Technology, 43600 Attock, Pakistan

## Abstract

Due to the proliferation of handheld mobile devices, multimedia applications like Voice over IP (VoIP), video conferencing, network music, and online gaming are gaining popularity in recent years. These applications are well known to be delay sensitive and resource demanding. The mobility of mobile devices, running these applications, across different networks causes delay and service disruption. Mobile IPv6 was proposed to provide mobility support to IPv6-based mobile nodes for continuous communication when they roam across different networks. However, the Route Optimization procedure in Mobile IPv6 involves the verification of mobile node's reachability at the home address and at the care-of address (home test and care-of test) that results in higher handover delays and signalling overhead. This paper presents an enhanced procedure, time-based one-time password Route Optimization (TOTP-RO), for Mobile IPv6 Route Optimization that uses the concepts of shared secret Token, time based one-time password (TOTP) along with verification of the mobile node via direct communication and maintaining the status of correspondent node's compatibility. The TOTP-RO was implemented in network simulator (NS-2) and an analytical analysis was also made. Analysis showed that TOTP-RO has lower handover delays, packet loss, and signalling overhead with an increased level of security as compared to the standard Mobile IPv6's Return-Routability-based Route Optimization (RR-RO).

## 1. Introduction

Mobility management for IPv6-based nodes, or Mobile IPv6 [1], facilitates the mobile nodes to move from one IPv6 address to another by migrating active transport layer connections and application sessions. As per Mobile IPv6's specification [1], the concept of home agent (HA) is used that proxies the mobile node (MN) to a fixed permanent address, called home address (HoA). In case of roaming, mobile node establishes a bidirectional tunnel [2] with its HA using a local care-of address (CoA). A binding is maintained by the HA between a mobile node's home address and its care-of address and packets are forwarded to the mobile node's new IPv6 address (which is the care-of address) using a bidirectional tunnel, which are destined to mobile node's home address. Hence, mobile node becomes reachable at the new location and can so communicate.

**Figure 1:**
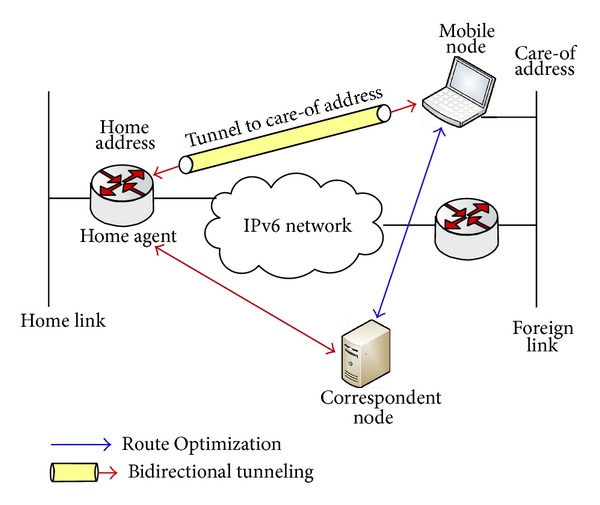
Mobile IPv6 bidirectional tunnelling and Route Optimization.

Using bidirectional tunnel, packets for MN travel through the bidirectional tunnel between the home agent and the mobile node. Inefficient route and increased overhead are the problems that occur due to encapsulation and tunnelling of packets through the home agent. The performance of real-time applications degrades, as inefficient route increases the end-to-end delay. Also, the header overhead due to encapsulation results in bandwidth inefficiency, and fragmentation may occur. A simulation analysis was made in [3] for the comparison of delay and overhead generated by Mobile IPv6's bidirectional tunnelling and Route Optimization. Results showed that the bidirectional tunnelling has higher end-to-end delay as compared to the Route Optimization. To overcome this problem, Mobile IPv6 proposed Route Optimization [1] that allows the two nodes to communicate using the direct path without tunnelling packets through the home agent. The basic principle of Route Optimization is to enable packets to directly reach the mobile node by avoiding tunnel through the home agent.  Figure 1 shows the basic operation of Mobile IPv6's bidirectional tunnelling and Route Optimization.

Route Optimization requires maintaining a binding entry between a mobile node's home address and the care-of address at the correspondent node (CN); hence, control signalling is done between the mobile node and corresponding node. The problem is how can the two end nodes (mobile and correspondent) authenticate and authorize the control messages that they exchange? From security viewpoint, to establish a binding entry at the correspondent node requires the verification of the mobile node for claiming of both addresses (home address and the care-of address). If this compulsion is compromised, then security threats like impersonation and flooding will arise [4].

**Figure 2:**
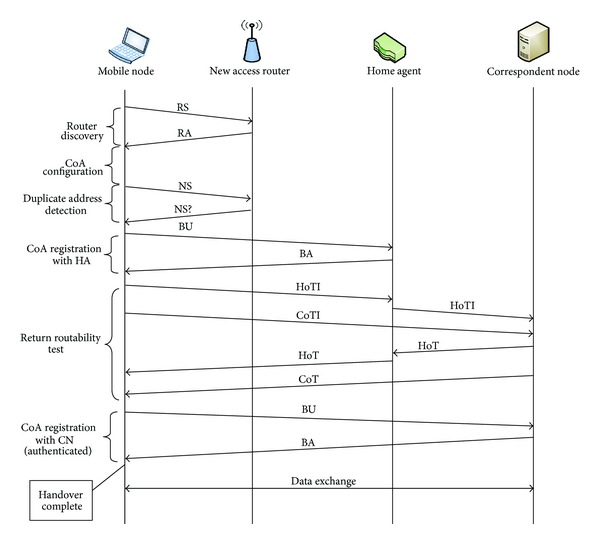
Mobile IPv6 Route Optimization using Return Routability sequence flow.

For the authentication of mobile nodes, Mobile IPv6 proposed Return Routability procedure.  Figure 2 shows the sequence of the message flow for the Mobile IPv6 Route Optimization with Return Routability. In this procedure, two different tests (home address test and care-of address test) are performed to check the reachability of the mobile node at the home address and care-of address, respectively. To perform the home test, the mobile node sends a home test init (HoTI) message to the correspondent node via the home agent which then responds with home test (HoT) message by the correspondent node, again via the home agent. In case of care-of address test, mobile node sends care-of test init (CoTI) message directly to the correspondent node and receives back the response, the care-of test (CoT) message without routing through the home agent. In case of successful verification at both addresses, legitimacy of binding between the home address and the care-of address is proved. Hence, the mobile node sends the Binding Update (BU) message to correspondent node directly without involving the home agent. The correspondent node acknowledges the message with Binding Acknowledgement (BA). Now, direct communication can be done between the mobile and correspondent nodes.

Return-Routability-based Route Optimization (RR-RO) has an advantage that it is a lightweight procedure and has no requirements of preshared authentication keys [5]. Also, it does not maintain states at the correspondent node. On the other hand, this process has an undesirable impact on handover delays and results in longer handover delay and increased overhead as it involves signalling among all the three nodes (MN, HA, and CN) involved in the mobility management. This is because the home address test and the care-of address test involve message exchange between the mobile node and the correspondent node, between the mobile node and home agent and between the home agent and correspondent node. The latency of home address test is high as compared to correspondent address test, as it routes the messages through the HA and mobile node cannot resume the communication until both tests are completed. The delay caused by these two reachability tests (home address test and care-of address test) may be undesirable for different real-time applications or interactive applications for example, applications, like Voice over IP (VoIP) video conferencing, and so forth [5].

Another problem of Return Routability procedure with respect to security is that the security assured by the Return Routability procedure might not be enough for applications that are security sensitive. The reason is that Return Routability is vulnerable where attackers can interject in the home or care-of address test. Ren et al. [6] have discussed problems with the security of Mobile IPv6 Route Optimization and explained how it is vulnerable to false Binding Update, man-in-the-middle, and denial of service attacks.

And finally, this procedure requires periodic signalling exchange in an interval of at most 7 minutes, even in the case when the mobile node does not move and does not change IP connectivity [5]. This periodic refresh of binding registration at the correspondent node involves additional signalling overhead.

So considering all above discussed problems, this paper proposes an optimization to Mobile IPv6 Route Optimization through a correspondent node compatibility test and new security mechanism. In the proposed solution, mobile node keeps record of Mobile IPv6 compatible correspondent nodes and in case of mobility, mobile node communicates only with those correspondent nodes that have an active Mobile IPv6 implementation. Also, the new proposed mechanism does not route the signalling messages via the HA (rather signalling is done directly) and does not involve the communication between the HA and CN, that results in further performance improvement.

The organization of the rest of the paper is as follows. In Section 2, motivation for the improvement of Mobile IPv6's Route Optimization has been discussed.  Section 3 discussed the related work and Section 4 presents the proposed mechanism of improved Route Optimization solution. In Section 5, evaluation of the proposed system has been carried out through simulation, security analysis and analytical analysis, and results have been discussed. Finally, conclusion is made in Section 6.

## 2. Motivation for Improvement of Mobile IPv6 Route Optimization

The IP-based multimedia and real-time data deliver are the main motivation for the enhancement of Route Optimization. Due to the proliferation of handheld mobile devices, multimedia applications like Voice over IP (VoIP), video conferencing, network music, and online gaming are gaining popularity in recent years. These applications are well known to be delay sensitive and resource demanding. Therefore, any attempt that tries to overcome the delay in the Internet at any point (from the mobile node to the correspondent node) will be greatly appreciable. Hence, an improved Route Optimization approach with less delay and overhead is apparently important and desirable for the Internet.

As wireless networks with frequent movements of nodes have a number of salient features that differentiate them from networks with stationary nodes or with nodes having very occasional movements, hence efficiency is an important aspect of mobility management in these environments. In the literature, attempts that are made to enhance Mobile IPv6's Route Optimization generally try to reduce the handover latency, to increase the security level, to lower the signalling overhead, and to increase the protocol robustness.

If a mobile node does not often change its position and IP attachment point, then the handover latency will not be a concern and there is no need to worry about a few round trip times (RTT) of delay. But with a higher level of mobility, application performance is compromised. Therefore, handover latency that is added by the mobility management protocol to the existing delays of the network should be reduced to increase the user satisfaction level.

In addition, data transmission in wireless environments creates security issues. It becomes easier for intruders and attackers to eavesdrop the data of others or to send data on some other nodes, behalf. Usually applications encrypt and authenticate their data if they need some security, but such security measure may not be feasible for mobility-related signalling messages if no preexisting relationship is there between the mobile and correspondent nodes.

Also, the bandwidth in the wireless medium is typically limited; hence, resources should be used in an economic way. In case of mobility management, this objective is relate to the amount of signalling messages that a mobility management protocol exchanges.

In this section, we have discussed the needs of optimization for each of the above issues.

### 2.1. Enhancement for Reducing Latency

An important objective of the Route Optimization improvement is to reduce the handover latencies. Mobile IPv6's care-of address test has lower latency as compared to the home address test, but mobile nodes cannot resume communication until both tests (HoA test and CoA test) are completed. Hence, the latency caused by the home address test will be considered for the overall handover latency.

Keeping above point in mind, in a Mobile IPv6 handover, home registration between the mobile node and home agent takes one RTT, the home address test takes one RTT to transfer HoTI and HoT between the mobile node and home agent plus an RTT between home agent and correspondent node for transferring the same two messages (HoTI and HoT), and also a one way time from the mobile node to the correspondent node for the propagation of the *Binding Update* message. The first data packet to be received at the mobile node's new location (new care-of address) takes additional one way time from the correspondent node to the mobile node. If the mobile node is sending data, then it resumes the communication right after sending the *Binding Update* message. If the mobile node has requested a *Binding Acknowledgement* message from the correspondent node, then mobile node will not resume communications until it receives an *Acknowledgement* message from the correspondent node. The four delays discussed here are additive and do not include the other delays at the IP layer or link layer.

The long handover latencies may result in significant quality degradation for applications that are interactive or real-time. Similarly, TCP data transfer in bulk form is also affected as long handover delay may lead to retransmissions, due to retransmission timeout (RTO) expiry, hence degrading the throughput.

### 2.2. Enhancement for Increasing Security

The objective of Mobile IPv6's Return Routability procedure was to provide a certain level of security that may be compared to the security level of today's nonmobile Internet [4]. The assumption of Return Routability is that mobile Internet cannot be as safe as the non-mobile Internet. Hence, it protects against certain attacks like impersonation, denial of service, redirection, and flooding. It is recommended for the applications which require much more security than provided by the Return Routability that they may use some end-to-end protection mechanisms like IPSec and so forth. But still they are susceptible to denial of service attacks. This problem motivates the research community to develop stronger security solutions for Route Optimization.

The main weakness in the original RR-RO is due to the nonsecure communication on the communication path of home agent and correspondent node. If this path is made secure, all intrusions against Mobile IPv6 Route Optimization can be protected by the original RR-RO protocol [1, 4].

As home and care-of addresses are carried in plaintext in the route optimized packets, hence location privacy is also an important issue with Route Optimization. To solve this problem, a standard way is to use the traditional bidirectional tunnelling whenever there is any need for location privacy. Thus, packets with the care-of address can be secured through IPSec Encapsulating Security Payload (ESP) encryption [7], as they will be transferred only between the mobile node and the home agent. But even with the use of bidirectional tunnelling, periodic reestablishment of IPSec security associations is performed through Security Parameter Indexes (SPIs) by the mobile node with the home agent so that it may become untraceable.

### 2.3. Enhancement for Signalling Reduction

Route Optimization in Mobile IPv6 exchanges periodic signalling messages even in the absence of mobile node's movement. This signalling overhead is calculated as 7.16 bps if the mobile node is communicating with a stationary correspondent node [8]. According to Arkko et al. [5], Route Optimization requires periodic signalling exchange in an interval of at most 7 minutes, even in the case when the mobile node does not move and does not change IP connectivity. These periodic refreshes messages consuming a fraction of the wireless bandwidth that is already limited and can be used more efficiently in the absence of these periodic messages. The amount of this overhead will be doubled in case both the communicating nodes are mobile. This overhead may be negligible as compared to mobile node's data traffic when the node is communicating, but in case when the node is in inactive state this may be an issue. As battery power is a limitation of mobile nodes, hence they usually prefer to be in standby mode to save battery power. Optimizations for reduced signalling overhead could overcome these issues.

### 2.4. Enhancement for Protocol Robustness

Home agent failure may happen or may become temporarily unavailable; hence, Route Optimization should enable the communicating nodes to continue communications during such periods. The Mobile IPv6 defined by the Perkins et al. [1] does not achieve this independence. This is because; the home agent plays an active role in the Return Routability procedure. Appropriate optimizations could result in a solution that is independent from the home agent and more robust.

## 3. Related Work

Some enhancements to the standard the Mobile IPv6 Route Optimization have been proposed in the literature. A detailed survey of security protocols for Mobile IPv6 Route Optimization has been done by Modares et al. [9]. We have considered some of them which are more relevant to the proposed work.

Perkins in [10] proposed an alternative security mechanism with low latency for protecting signalling related to the Route Optimization. This technique used pre-configured data for precomputing a shared secret *Kbm* (Binding Management Key) between the mobile node and its correspondent node. This precomputing of shared secret can subsequently be used for authorizing Binding Updates and removing the messages related to the routability tests, thus achieving significantly smaller latency. The proposed mechanism will simply overcome the problem of longer delay and overhead in case when both the communicating parties have an active implementation of Mobile IPv6. In case the correspondent node is not Mobile IPv6 aware, then the mobile node has no information about that and it will be using the home address test and care-of address test, creating overhead traffic that is of no use and corresponding node will always continue sending data to the home agent.

Le and Chang [3] have proposed ROT (Route Optimization Tunnelling), an alternative routing enhancement mechanism based on end-to-end tunnelling extension to Mobile IPv6 for data packet routing. The authors suggested using tunnel header instead of Type 2 Routing header and Home Address option to carry both the MN's and CN's home addresses. For a received packet, to decide whether to route the packet to CoA directly by the standard routing mechanisms, or to use the proposed mechanism, the format of the *Binding Update* messages is modified and an ROT flag is added in the header to indicate that the endpoint that sends the *Binding Update* messages is using ROT. Upon receiving an extended Binding Update message, the ROT-aware correspondent node should utilize the ROT Tunnel Manager to process any incoming and outgoing data packets. Any correspondent node that is ROT-unaware will simply ignore the bit and perform normal Mobile IPv6 operations. The proposed solution attempts to avoid the bidirectional tunnelling and proposed a mechanism just for data forwarding, without considering that in standard Mobile IPv6's Route Optimization is using the Return Routability procedure for the security purpose and that is the main concern for the delay and overhead in existing Route Optimization. The authors have not given any details about how to authenticate the mobile node and to secure the direct control communication between the mobile node and the correspondent node. If the proposed mechanism is still using the Return Routability for authentication of mobile nodes, then the overhead and delay that Mobile IPv6's Route Optimization was facing are still there.

Deng et al. in [11] proposed the Certificate-Based Binding Update protocol (CBU) that uses the public private key pair and digital signature cryptosystem. This system is dependant on a certification authority (CA) for the issuance of certificates to nodes. Thus, depending on additional network entities for authentication purpose may lead to unnecessary delay.

Ren et al. [6] proposed a Hierarchical Certificate-Based Binding Update (HCBU) protocol with high security strength. It makes use of public key certificate-based strong authentication technique. Three-layer hierarchical trust management framework is introduced in order to solve the difficult certificate issuing and management problem of [11]. The CA is required to issue certificate to only Tier-1 ISPs, which minimizes the number of required certificates managed by CA(s). The Binding Update protocol authenticates the mobile node's home address and care-of address to CN via the MN's home agent. Rena et al. suggested that one of the home agent's functions is to act as a security proxy for its mobile nodes. The authentication is based on the home agent's certificate and the secret session keys are generated by strong cryptosystems. Many security problems of Return Routability have been overcome, but the problem of delay is still there as Binding Updates will be performed via the home agent. These proposals [6, 11] focused on the procedures time and security enhancements to the Return Routability. They did not consider the signalling optimization.

A new secure and lightweight Route Optimization procedure for network mobility (NEMO) has been proposed in [12]. The idea is based on exploiting the firewall to perform Route Optimization services on behalf of the correspondent nodes. The firewall acts as an agent for the correspondent nodes without modifying them, thus supporting Route Optimization in NEMO. The proposed mechanism provides secure communications by making an authorized decision about the mobile router's (MR) home address, care-of address, and the complete mobile network prefixes underneath the MR. In this enhanced protocol, the MR can extend the HoTI message to include one or more selected numbers of the network prefixes underneath the MR. Following this new mechanism, the MR will first check whether its own listed prefixes are able to provide the RO. This verification will occur depending on the flag bit added to the mobile header by the MN, to inform the MR if the MNN is sophisticated enough for the RO. This flag is called the “z” flag. When the flag is set to “on,” it means that the MNN is sophisticated enough for the RO; otherwise, the flag is set to “off.” This flag is added to the MR prefixes list for verification purposes. The MR will send the extended mobility header with a list of prefixes that are able to function under the RO to the HA_MR using the HoTI message. On the other side of the network, the modified firewall protects either the CN or the corresponding router (CR) in the correspondent network. The firewall uses newly developed stateful filtering rules that allow the packets destined to or from the HA or MNN to pass to or from the CN network, without filtering the data messages and the signalling.

The idea [12] is for network mobility and not for a mobile node's mobility; even if it is extended for mobile node's mobility, it requires implementation of the new entity firewall. Also the mechanism is still using the Return Routability signalling of home test and care-of test that are the major problems in the protocol's overhead and delay.

Ali Alsalihy and Alsayfi [13] have proposed Return Routability Identity-Based Encryption (RR-IBE) protocol for the enhancement of security and authentication in the original Mobile IPv6's RR-RO protocol. This protocol requires a third party, Private Key Generator (PKG), for the distribution of keys. The keys are P and s.P. The third party simultaneously distributes the key to all senders (CNs). This PKG maintains the private keys for the CN. MN sends a packet *P*1 to CN via the HA and also a packet *P*2 to CN directly. Both *P*1 and *P*2 contain encrypted messages that have a random number r0 as well. The CN compares the contents of *P*1 and *P*2 without encrypting them. If both messages are similar, the CN will make a request (messages *P*3 and *P*4) from the appointed PKG to obtain its own (CN's) private key and to decrypt one of the messages (*P*1 or *P*2). After decryption, if the contents of both *P*1 and *P*2 are same, the CN generates a random number *r*1, composes the message by XOR on *r*0 and *r*1, and sends packet *P*5 to the MN for authentication purpose. If *P*5 is correct, the MN generates a random number *r*2, generates the messages by XOR on *r*1 and *r*2, and replies to the CN with packet *P*6. Finally, to ensure that the authentication is correct, the CN will reply with packet *P*7 to the MN. After the mutual authentication between CN and MN is completed, both are able to send and receive messages.

The RR-IBE attempted to overcome the problem of security issues of normal RR-RO protocol, but it introduces the network entity PKG and requests for private key every time a handover is executed. Also the first message *P*1 is sent via the home agent. These two options cause delay and overhead in the handover process.

Reference [14] proposed the improved Tunnelling-Based Route Optimization as an extension of the work of Le and Chang [3]. According to [14], the route optimized tunnel overhead can be reduced more when both nodes are mobile. It is assumed that traditional Route Optimization signalling with Return Routability has been performed and a tunnel is already initiated between mobile and correspondent nodes along with exchange of Binding Updates from both nodes to construct the Binding Cache entries for HoA-CoA pair at the other end. When the Tunnel Manager gets the packet from Network layer, it changes the source address field to MN's CoA and looking into the Binding Cache changes the destination address filed to the CN's CoA. Hence, there is no need to send the HoA of the other pair via header extension because it can be obtained from the Binding Cache with the help of CoA included in packet.

The proposal [14] tried to decrease the overhead that is caused for the data transmission after the Route Optimization signalling has been done. The problem with the original RR-RO is the overhead and delay involved during the signalling of control messages and not for the data transmission. The control overhead in the data packet is negligible as we look into the size of data packet. Hence, this technique cannot be considered as the alternative of RR-RO.

Susanto and Kim [15] proposed two Per-Connection RR Test Schemes. Each scheme performs normal Return Routability test at the beginning of a connection between mobile node and correspondent node and eliminates per-handoff Return Routability test in subsequent handoffs. Hence, it attempts to reduce the delay involved in the Return Routability test in each handoff. One of the schemes, Authentication Pool scheme, uses a pool of encrypted tokens that are sent from mobile node to correspondent node. After that, when a mobile node is ready to perform the *i*th handoff, it generates a new binding key so that Kbm^*i*^ = SHA1(TK*i*, M), where M = (previous CoA∣HoA∣TK*i*-1). The encryption key Kbm^*i*^ is derived from the previous CoA and the previous token of TK*i*-1. Upon receiving a new *Binding Update* message, correspondent node derives the encryption key used for the *Binding Update* message from the previous CoA, HoA, and the previous token of TK*i*-1. This allows correspondent node to be able to decrypt the BU message for the handoff. In this authentication pool scheme, mobile node generates a set of tokens, which is included in the *Binding Update* message. In case pool is large, the size of *Binding Update* message may be increased, resulting in slightly longer transmission time of the initial *Binding Update* message. The initial *Binding Update* message also contains the pool of tokens that may be intercepted by the adversary. Although the *Binding Update* message is encrypted, still there may be concerns that it may be compromised.

The second functionality of [15], Token scheme used the one-time password to create a hash chain of authentication tokens. This method allows the authentication to be performed without exchanging or distributing information over the network. Also, the amount of information required to be maintained at CN is kept to its minimum.

The protocols discussed so far in this section either focused on the security aspects of Route Optimization or reduced the overhead of existing Return Routability procedure. In comparison to the literature, the proposed TOTP-RO protocol attempts to reduce the delays and overhead involved in the Route Optimization procedure along with focusing on the authentication of mobile node and to avoid the certain security attacks that can be launched on the Route Optimization procedure.

## 4. Enhanced TOTP-Based Route Optimization (TOTP-RO) Procedure for Mobile IPv6

In the proposed time-based one-time password Route Optimization (TOTP-RO) enhancement, two functionalities are used. In the first phase, at the start of communication signalling is done between the mobile node and corresponding node to check the compatibility of correspondent node for Mobile IPv6 implementation and for the calculation of shared secret *Token*. In the second phase, a mobile node communicates with correspondent nodes directly and does not perform signalling like home test and care-of test which are performed in the Return Routability procedure of route optimized Mobile IPv6.

To secure the communication, the concept of shared secret *Token* along with *One-Time Token* is used. All the protocol messages in the proposed TOTP-RO are carried within the IPv6 Mobility Header [1]. The following notations have been used in this Section:

 
*k*_*i*_: The ECDH public key for node *i*
 CCRE: Complex Conversion Routine Encoding
 P128: Compression permutation function
 K: Shared secret key
 Token: Shared secret token
 SHA: Secure Hash Algorithm 1
 
*X*_*i*_: Random number at node *x*
 OTT: One-Time Token
 TOTP: Time-Based One-Tome Token
 MD5: Message Digest 5.

### 4.1. Shared Secret Token Computation

In the proposed enhancement scheme, a shared secret identifier (*Token*) is used to achieve the security. This *Token* is computed through the use of an Elliptic Curve Diffie Hellman (ECDH) key exchange at the start of communication between two end nodes. The proposed mechanism does not depend upon any third party to authenticate the *Binding Update* messages that are sent to the correspondent node, thus permitting the end nodes to use the authentication method which they prefer for the establishment of trust relationship.

The computation of shared secret Token is achieved just like it is done by Snoeren and Balakrishnan [16]. For any node *i*, which is initiating the communication (initiator), the generation of its ECDH public key *k*_*i*_ is done by selecting a random number and then encoding using the complex conversion routine encoding (CCRE).

The random number is

(1) Xi={1,2,3,…,n−1},

where *n* is the order of *a*, *a* is a number for which *a* ^*n*^ = 1(*modp*), and *p* is a prime number. Now, computing the ECDH public key for node *i* is as follows:

(2) ki=P128[CCRE(Xi∗a)],

where *P128* is a compression permutation function that produces the 128 least significant bits as output for the input given to it. The ∗ is the traditional mathematical multiplication operator. The final *k*_*i*_ is a *17-Byte* value that will be sent to the other node (responder).

When the respondent node *j*, with a compliant Mobile IPv6 implementation, receives message from initiator node *i* with ECDH information, it selects the random number *X*_*j*_ = {1,2, 3,…, *n* − 1} in the same manner as done by node *i* and uses it to construct its own ECDH public key *k*_*j*_ and sends back to the initiator node:

(3) kj=P128[CCRE(Xj∗a)].

Now, both nodes have the same set of public keys and can compute the shared secret *Token*. First, they compute the shared secret key *K* as

(4) K=ki∗Xj=kj∗Xi.

Now, the shared secret *Token* can be computed from *K* that will be used for authentication and validation in case of Route Optimization during mobility management. The computation of the *Token* is accomplished by concatenating the key (*K*) and the random numbers *X*_*i*_ and *X*_*j*_ and then computing the hash using the Secure Hash Algorithm (SHA-1):

(5) Token=SHA(Xi,Xj,K).

The SHA is the SHA-1 algorithm that produces a unique 20-Byte *Token* value for the two particular nodes. The security of *Token* depends upon the values of *X*_*i*_ and *X*_*j*_; hence, they should be randomly generated.

**Figure 3:**
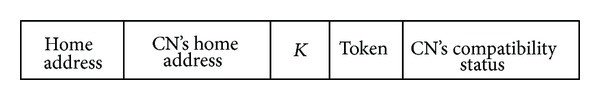
CN compatibility list format.

Now, both nodes have computed the same *Token* and made an entry in their *CN compatibility list*. It is a list, maintained at all the Mobile IPv6 compatible nodes and consists of home addresses, CN IP address, shared secret key *K*, *Token* and CN compatibility status of the nodes which are communicating to this particular node and is shown in Figure 3.

### 4.2. Correspondent Node's Compatibility Test

At the start of communication on initiator side, a node sends a message which we call *Node's Status Request *(NS_REQ), to check whether the correspondent node has support for Mobile IPv6 or not. This message requests from the responder (correspondent node) whether it has any support for Mobile IPv6 or not. NS_REQ also contains public key information to compute the *Token* and then to secure the control communication for TOTP-RR between two communicating nodes. The mechanism of shared secret Token computation is explained in the previous subsection. When the correspondent node (responder) receives this message, it can process it in two different ways as follows.

1. Responder node also supports Mobile IPv6, computes the *Token* and responds back to initiator by sending the *Node's Status Response* (NS_RES) message informing the mobile node that correspondent node also supports Mobile IPv6. When an initiator receives NS_RES from a correspondent node (responder) with public key information, it also computes the *Token*, and records the status of correspondent node as having support for Mobile IPv6 in the *CN compatibility list*. This status is a binary value and stores either True or False. The *Token* that is computed at both ends is the same and will be utilized to secure the future control communication for Route Optimization between mobile and correspondent nodes. Details are explained earlier in shared secret Token computation subsection.
2. If the correspondent node (responder) has no support for Mobile IPv6, then it does not know NS_REQ message. CN discards the message and an ICMPv6 error message is returned. When ICMPv6 error message is received, then the mobile node (initiator) records the correspondent node's status as it has no support for Mobile IPv6 in *CN compatibility list*. In this case, no Route Optimization will work, and the mobile node will not communicate with the correspondent node, rather bidirectional tunnelling will always be used.

This correspondent node's status which is maintained at the mobile node is used for TOTP-RO during handover management. Details are discussed in the next *Enhanced Route Optimization* subsection.

**Figure 4:**
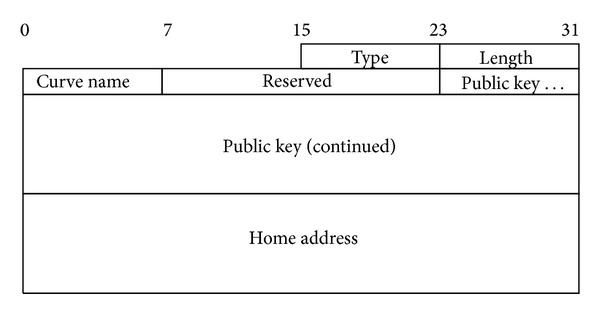
Node status request and response messages header format.

All the TOTO-RO messages are carried in the *Message Data* field of Mobility extension Header of base IPv6 header, which is identified by the Next Header value of 135 [1]. The header format of NS_REQ and NS_RES messages is as in Figure 4, where Type is the type of message whether *Node's Status Request* or *Node's Status Response*. NS_REQ has Type = 24 and NS-RES has Type = 25, Length is the length of the message which is 38 Bytes, *Curve Name *is the name of the curve used for ECDH algorithm, *Reserved* is the field reserved for future use, *Public Key* is the 17-Byte ECDH public key for that node, and *Home Address* is the IPv6 home address of the node sending the message.

### 4.3. Enhanced Route Optimization

After receiving a new router advertisement message from access router, mobile node acquires a new IPv6 address and detects its movement into the new network using movement detection mechanism [1]. Mobile node checks the correspondent node's compatibility for Mobile IPv6 in the *CN compatibility list*, meaning whether correspondent node supports Mobile IPv6 or not.

**Figure 5:**
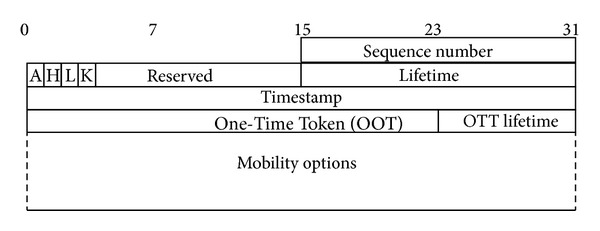
Modified Binding Update header format.

In case correspondent node also supports Mobile IPv6, then mobile node interacts with correspondent node directly and sends *Modified Binding Update *(MBU) message. The *Modified Binding Update* message contains the authentication information in addition to normal Mobile IPv6's *Binding Update* information. This authentication information includes a One-Time Token (OTT) and *Timestamp*.  Figure 5 shows the header format for *Modified Binding Update* message. The OTT is generated using the time-based one-time password (TOTP) technique [17] by concatenating the shared secret *Token*, mobile node's home address (HoA) and CoA, and the *Timestamp* as below:

(6) OTT=TOTP[MD5(Token ∣ HoA ∣ CoA ∣ Timestamp)].

The MD5 function generates the message digest and the TOTP function uses digested text to first generate a binary code using the following algorithm [17]:

 int offset = digested_text [16] & 0xf;
 int binaryCode = (digested_text[offset] & 0x7f) << 24
 
*|* (digested_text [offset + 1] & 0xff) << 16
 
*|* (digested_text [offset + 2] & 0xff) << 8
 
*|* (digested_text [offset + 3] & 0xff);

And then the binary code is converted to 6-digit decimal code using

 int ott = binaryCode % (int)pow(10, 6);

The *TOTP* function generates a unique 6-digit decimal number each time whenever a *Modified Binding Update* message is needed to be sent. The 6-digit decimal value is then converted to 24-bit (3 Bytes) binary value. Correspondent node upon receiving the *Modified Binding Update* message from a mobile node, with *OTT* and *Timestamp *information, computes its own *OTT* taking the home address and *Timestamp* from the received packet and shared secret *Token* value from its local *CN compatibility list*. Correspondent node then verifies the message by comparing the computed *OTT* value with the received *OTT* value. In case both *OTT* values are matched, correspondent node updates the *Binding Cache* with a binding entry for mobile node's home address and care-of address and sends *Binding ACK*, if the Acknowledge (A) bit is set by the sending mobile node to request a *Binding Acknowledgement*.

The home address is not communicated through the *Modified Binding Update* header; rather it is mentioned in the Home Address option which is carried in the Destination Option extension header in the base IPv6 packet. Also, the care-of address in not explicitly mentioned in the *Modified Binding Update* message and it is taken from the source address field of the received IPv6 packet.

After successful *Binding Update*, correspondent node sends data to the mobile node's new location (care-of address) directly using Type 2 Routing header [1].

All the fields in the Modified Binding Update are the same as used in the standard Binding Update except the Timestamp, One-Time Token and *OTT Lifetime*.

*Timestamp* is the timestamp, *One-Time Token *is a 3-Bytes field that contains the *OTT* value and *OTT Lifetime* is the lifetime for the OTT value contained in preceding field.

This procedure decreases the handover control signalling as compared to standard Mobile IPv6 Route Optimization with Return Routability, where control signalling is also performed between the mobile node, the home agent, and correspondent node for home test (*HoTI* and *HoT*), between the mobile node and the correspondent node for care-of test (*CoTI* and *CoT*) and between the mobile node and the correspondent node for *Binding Update* and *Binding ACK*. Thus, proposed TOTP-RO solution achieved the care-of address registration at the correspondent node through *the Modified Binding Update* and *Binding ACK* messages only in a secured way.

In case the entry in the local *CN compatibility list* for correspondent node is 0, then it means that correspondent node has no support for Mobile IPv6. Here, Route Optimization will not work and the mobile node will not send any *Modified Binding Update* to the correspondent node. Hence, the mobile node has to continue communication through bidirectional tunnelling via the home agent. In this case, the mobile node sends *a Binding Update* to home agent only. Now, the incoming data from the Internet for mobile node will be received by the home agent first, which is then forwarded to the mobile node's new location using the care-of address in a bidirectional tunnel. This optimization mechanism once again reduces the control traffic by not performing any control signalling with the correspondent node and the home agent for Return Routability procedure which is performed in the standard Mobile IPv6 Route Optimization.

**Figure 6:**
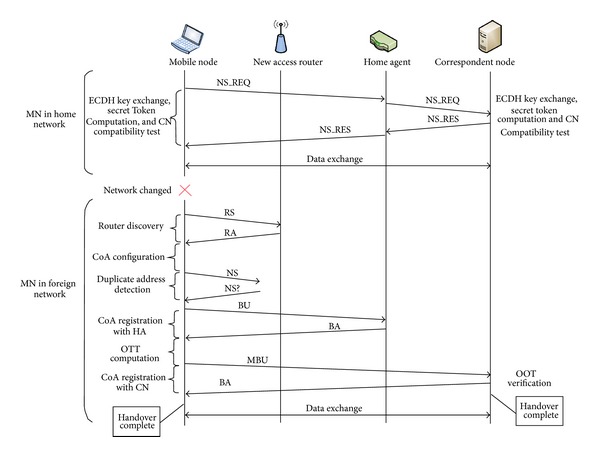
Proposed enhanced TOTP-RO sequence flow.

To achieve signalling optimization, in the proposed TOTP-RO, the Route Optimization signalling is performed only once for a handover unless there is any need to authenticate the other party. Also, the mobile node does not send periodic *Binding Update* messages to correspondent nodes or home agent, rather each time when a binding lifetime entry is expired the node checks for any traffic for which there exists a binding entry in *Binding Cache*. In case there is traffic for the *Binding Cache* entry, the node simply resets the lifetime to the previous initial lifetime value, thus reducing the overall signalling overhead. The home agent and correspondent node also do the same functionality before deleting any binding entry for which the lifetime has expired. If no traffic is observed for that particular binding entry, then the *Binding Update* may be performed with zero lifetime value.  Figure 6 shows the sequence of message flow for the proposed enhanced TOTP-RO.

## 5. Evaluation

In this section, evaluation of the proposed enhanced TOTP-RO has been carried out. The evaluation is done using simulation, security analysis, and analytical analysis.

### 5.1. TOTP-RO Simulation Analysis

The proposed solution for Mobile IPv6 Route Optimization was simulated in network simulator 2 (NS-2.33) by making changes in the Mobile IPv6 implementation of RFC 3775 in the MobiWan patch [18].

**Figure 7:**
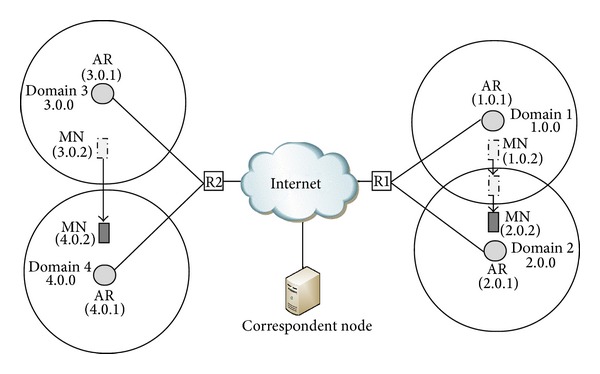
Network topology for mobility simulation.

Simulations were performed with different numbers of mobile nodes communicating with a single correspondent node with the following settings: Channel type: Wireless Channel, Propagation model: Two Ray Ground, Network interface type: Phy/Wireless Phy, Mac type: Mac/802_11, Interface queue type: DropTail/PriQueue, Link layer type: link layer (LL), Antenna model: Antenna/Omni Antenna, Interface queue length: 50, and Adhoc routing protocol: adhoc on-demand distance vector (AODV). The simulation model is presented in Figure 7.

The 802.11 WLAN was used as the wireless access network with the data rate of 2 Mbps. There were 4 Access Routers (AR), each of them has WLAN Access Point functionality. Each AR has radio coverage of 250 m and the region of overlapping coverage between the adjacent cells (Domain1 and Domain2) was 50 m. The mobile nodes moved within an area of 1600 × 1600 m with the constant velocity of 1 m/Sec. The number of mobile nodes and the traffic load were kept changing in different simulation setups to verify the correctness of proposed optimization technique. All the 4 wired links between ARs and the Internet backbone routers (R1 and R2) have 2 Mb of data rate with a delay of 2 msec. The wired Internet link between the two backbone routers (R1 and R2) has the data rate of 100 Mb and a delay of 10 msec.

**Figure 8:**
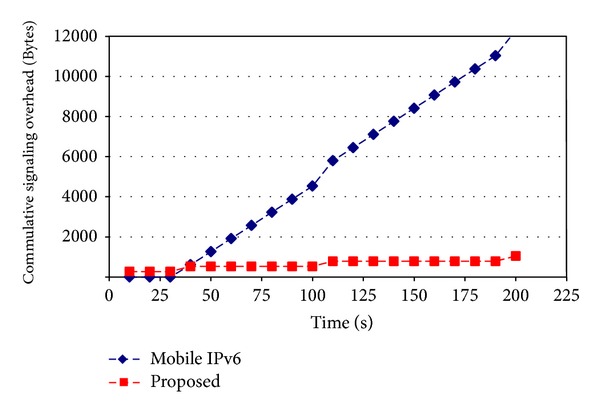
Signalling overhead comparison.

Figure 8 shows the signalling overhead comparison of Mobile IPv6 Route Optimization and the proposed scheme. It shows that the overhead produced by Mobile IPv6 is increased with time as Mobile IPv6 exchanged periodic binding refresh messages, but in the case of proposed solution the overhead remained constant as long as there was no movement of mobile nodes. When mobile node moved and performed some signaling, then the overhead for proposed solution was increased.

**Figure 9:**
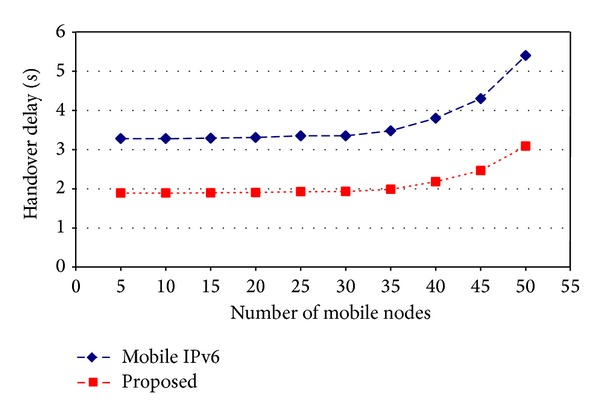
Handover delays comparison.

Figure 9 shows the handover delay comparison of Mobile IPv6 with the proposed technique. Handover delay has been computed with varying numbers of mobile nodes. Result show that the handover delay of Mobile IPv6 increased too much in comparison to the proposed technique as the number of mobile nodes increased. The reason for this reduced handover delay of the proposed technique was that proposed idea has not performed signalling like home test and care-of test as they were performed in the standard Mobile IPv6 Route Optimization.

**Figure 10:**
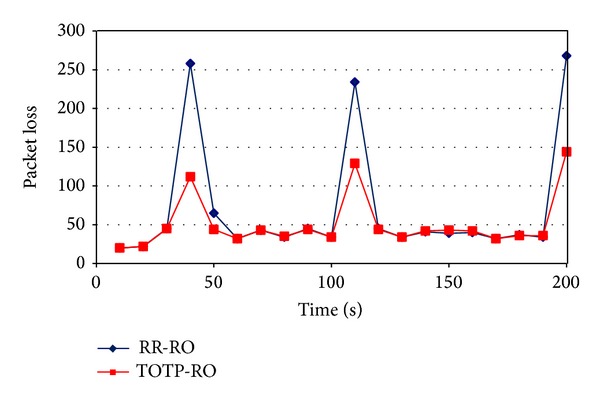
Packet loss comparison as a function of time.

Packet loss comparison is made in Figure 10. It can be observed at time *t* = 40, 110, and 200 seconds, that was actually the times when a mobile node undergone through handover, the packet loss of Mobile IPv6 was higher as compared to the proposed technique. It is because the handover delay of Mobile IPv6 is higher and packets are lost for a long duration of time as compared to the proposed optimization technique where the handover is completed early and resulting in less packet loss. In durations where there was no mobility, the packet loss of both techniques is almost same.

**Figure 11:**
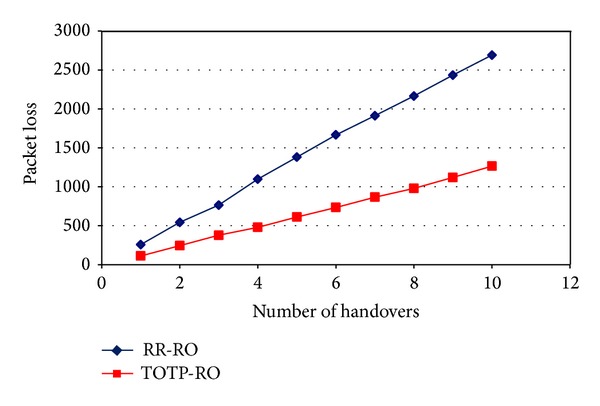
Packet loss comparison as a function of number of handovers.

Similarly, Figure 11 shows the packet loss comparison as a function of the number of handovers a mobile node undergoes during the simulation time. As mobile node undergone more and more handovers, the packet loss was increased for both mechanisms. The packet loss of Mobile IPv6 was much higher as compared to the proposed Route Optimization.

It can be observed from the results that the proposed Route Optimization has significantly reduced the signalling overhead, handover delay, and packet loss during the overhead. Route Optimization procedure in Mobile IPv6 involves the home address test and care-of address test that results in higher signalling overhead, handover delay and packet loss during the handover. The proposed mechanism for Route Optimization performs a direct *Binding Update* to the correspondent node and the authentication of the mobile node is done through secret *Token* and *OTT* generated using TOTP mechanism, hence resulting in less handover delay and less signalling overhead for handover.

### 5.2. TOTP-RO Security Analysis

In this subsection, the security strength of the TOTP-RO mechanism is analyzed. To analyze the security of the proposed solution, we have used the levels of power control that an adversary can gain [19].

 Level 1 control (basic sniffing or replay): the adversary can eavesdrop the traffic and can replay the message or gets the secret keys.
 Level 2 control (malicious message spoofing): the adversary can spoof a valid IP address and can generate malicious messages.
 Level 3 control (interception and modification): the adversary gets the full control of the communication and can modify the intercepted messages.

The basic design principle of RR-RO protocol assumes that an adversary who can monitor the mobile node-correspondent node path cannot monitor the home agent-correspondent node path. This assumption is a fundamental flaw of the protocol. There is no such reason to assume that an adversary will monitor one link and not the other especially when adversary knows that monitoring a given link is particularly effective to launch attack. So this assumption is not being used by the TOTP-RO.

One the other hand, the assumptions regarding the security of paths between different nodes which are made for the original RR-RO security are used in our security analysis as well. These assumptions are as follows: the path between the mobile node and the home agent is secure and protected by the IPSec ESP tunnel [7] and the paths between mobile node-correspondent node and home agent-mobile node are unprotected.

The behaviour of protocol in different security attacks with respect to the level of power control has been discussed below.

#### 5.2.1. Replay Attack

A replay attack is a form of network attack in which a valid data transmission is maliciously or fraudulently repeated or delayed. This is carried out by an adversary who intercepts the data and retransmits it later on.

As Route Optimization requires signalling of control message between mobile and correspondent nodes, on the basis of which traffic is redirected to mobile node's new location, hence an adversary can make a copy of the message and can launch a replay attack later on.

In the first case, if an intruder replays the MBU message without modifying its contents, then the correspondent node checks whether the OTT lifetime has expired or not. In case the OTT lifetime has already expired, correspondent node simply discards the message. If the OTT lifetime has not expired, then correspondent node checks for binding entry for that mobile node in the Binding Cache. In case of the replayed packet for which OTT lifetime has not expired, there will be a binding entry for that mobile node. Again, the correspondent node will simply discard the message.

In the second case, if an intruder tries to make changes in the MBU message, then the computed OTT will not match the received OTT and will not be verified; hence, an adversary will not be able to get the control.

#### 5.2.2. Data Confidentiality

Data confidentiality means the protection of data from unauthorized disclosure. As the MBU message contains no confidential data like secret key and so forth, hence there is no need for data confidentiality. For applications that require data confidentiality, IPSec ESP [7] can be used.

#### 5.2.3. Man-in-the-Middle Attack

In the “man-in-the-middle” (MITM) attack, an active attacker can listen the communication between two parties and also is able to change the contents. To execute this attack, the adversary pretends to be one of the parties in front of the other party by intercepting the messages in a public key exchange. The adversary then retransmits the public key messages, substituting his own public key for the requested one. The two original parties seem to be communicating with each other but in reality they are not.

In TOTP-RO, if an adversary pretends to be the mobile node to correspondent node and sends the false MBU message, then correspondent node while calculating the OTT value will get the CoA from the Source IP address field of the received IP packet, which is the address of mobile node and not of the adversary. Hence, OTT value will not be verified and the communication will not be directed to the adversary. Thus, the adversary will loose the MITM control over communication.

#### 5.2.4. Amplification Attack

Amplification is a reflection attack in which an adversary delivers traffic to the victim by reflecting it off of a third party so that the origin of the attack is concealed from the victim and the byte count of traffic received by the victim is substantially greater than the byte count of traffic sent by the adversary. In practice, this attack amplifies or multiplies the sending power of the attacker.

**Figure 12:**
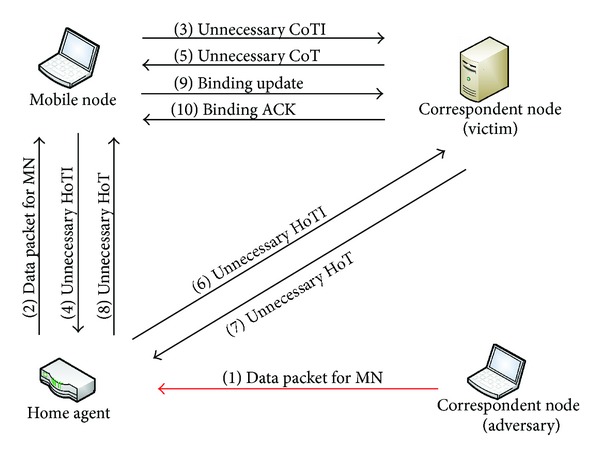
Amplification attack on RR-RO.

In the RR-RO, the amplification attack can be launched by sending a message to the mobile node through the home agent from the correspondent node to ask the mobile node to send more messages to another node, called the victim node.  Figure 12 shows the scenario of amplification attack for Mobile IPv6 RR-RO. The mobile node has moved to a new care-of address and an adversary sends a message to the mobile node on the behalf of a correspondent node (victim) by spoofing victim's address. The mobile node's home agent will forward the message to the mobile node's care-of address through the bidirectional tunnel. Upon receiving such message from a correspondent node via the home agent, mobile node triggers the Return-Routability-based Route Optimization that results in amplification of valid message exchange resulting in an increased overhead.

On the other hand, this type of attack is prevented in the TOTP-RO as there is direct communication between the mobile node and correspondent node and in case a message is received from an adversary via the home agent, the mobile node will send a single message, the MBU message, to the correspondent node. The correspondent node will check the Binding Cache and will definitely find an entry for the said mobile node; hence, message received will simply be discarded without generating additional signalling.

#### 5.2.5. Nonrepudiation

Nonrepudiation refers to the ability to ensure that a party to a communication cannot deny the authenticity of their signature on a document or the sending of a message that they originated. TOTP-RO uses the one-time password based digital signature for nonrepudiation. Hence, in case of successful verification of OTT value, it is confirmed that the MBU message has been received from the authenticated mobile node.

#### 5.2.6. Data Integrity

Data integrity refers to the fact that data has not been changed while in transmission. The TOTP-RO tries to secure the security information and not the entire payload; hence, other solutions can be used for data integrity. The integrity of security related information is verified from the received OTT. If the received OTT is verified, then integrity of control data is assured, meaning that the control information has not been changed in-flight.

#### 5.2.7. Denial of Service Attack

In denial of service (DoS) attack, an explicit attempt is made by the adversary to make a computer resource unavailable. This is achieved through either injecting a computer virus or flooding the network with useless traffic.

**Figure 13:**
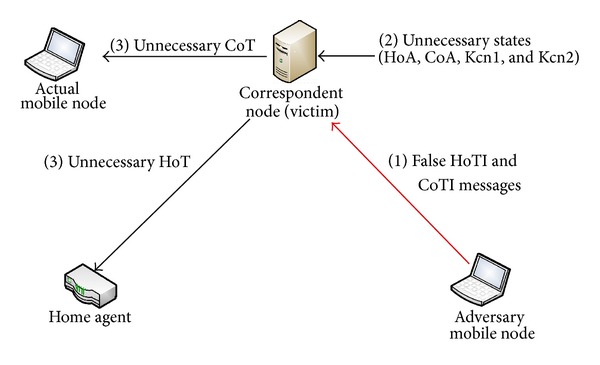
DoS attack on RR-RO.

This can be launched in the Mobile IPv6 Route Optimization by sending valid messages repeatedly. Due to maintenance of binding in Mobile IPv6, this makes it a stateful protocol; hence, it exposes the risk of DoS attacks. If a state is stored at a node which is the result of an unauthenticated message, then an adversary can initiate many such messages and might cause the node to run out of resources storing a large number of unnecessary protocol states. Particularly applying this problem of DoS to the *Binding Update* of mobile IPv6 is shown in Figure 13. The adversary sends the false HoTI and CoTI messages with fake home and care-of addresses, respectively, to the correspondent node (victim). The correspondent node will respond with two randomly chosen secret values, and it keeps these values until it receives the authenticated *Binding Update*. The adversary may repeat the generation of fake HoTI and CoTI messages many times and the victim (correspondent node) may run out of resources and will not be able to store all the state data resulting in drop of valid messages. This may prevent legitimate mobile nodes from using the Route Optimization with the correspondent node. The attack is similar to the SYN-flooding attack against the TCP protocol.

The TOTP-RO does not store any state for the mobile node until it is verified as an authenticated mobile node. False MBU messages will simply be discarded without maintaining any state, thus limiting the resource occupying.

#### 5.2.8. Redirection Attack

Redirection is a type of session hijacking where an adversary redirects the traffic to himself or to another node by sending a forged *Binding Update* message or replaying an older one and claiming that the mobile node has moved to this new care-of address location. This may redirect the traffic from the correspondent node to the adversary or may result in flooding of another victim node whom care-of address has been provided in *Binding Update*.

TOTP-RO while calculating the OTT value takes the care-of address value from the Source IP address of packet received; hence, for a forged *Binding Update* message, the OTT value may not be verified and redirection cannot be done. Even in case of IP spoofing, the computed OTT value will not match the received OTT value as the shared secret *Token* value is not known to the adversary; the redirection is not possible.

Table 1 shows the comparison of different Route Optimization protocols for their strengths and weaknesses against different security features.

### 5.3. TOTP-RO Analytical Analysis

This subsection analytically evaluates the proposed TOTP-RO mechanism and compares it with the original RR-RO. The following notations have been used in the analysis:

 
*T*_MN-HA_: The RTT on the mobile node-home agent path protected by the IPSec ESP tunnel
 
*T*_MN-CN_: The RTT on the mobile node-correspondent node path
 
*T*_HA-CN_: The RTT on the home agent-correspondent node path
 
*L*_*X*_: The handover latency for protocol *X*
 MAX: The maximum value of the 2 input values
 
*T*_*I*_: The RTT Internet time between two nodes communicating over the Internet
 
*T*: A given time interval for which mobile node does not move again
 
*t*: Binding lifetime
 
*x*: Number of times mobile node found the traffic for the particular binding entry for which binding lifetime has expired
 
*N*_*X*_: The number of messages exchanged by protocol *X* when the binding lifetime expires.

To achieve security, the original RR-RO protocol needs to be executed fully for every *Binding Update*. Thus, the latency of each *Binding Update* is approximated as

(7) LRR-RO=MAX[TMN-CN,(TMN-HA+THA-CN)] +12TMN-CN.

The first term in the MAX represents the delay in care-of test and the second term represents the delay incurred in home test. It is obvious that the home test will take longer time to complete as compared to the care-of test due to routing of messages via the home agent; hence, the latency caused by the original RR-RO protocol will be the delay for execution of care-of test plus one-way delay for the data packets on the direct path from correspondent node to the mobile node.

The latency incurred by the TOTP-RO protocol is approximated as

(8) LTOTP-RO=TMN-CN(1+12).

Let *T*_*I*_ represents the one RTT Internet time between two nodes communicating over the Internet and assume that all the RTTs for the mobile node, the correspondent node, and the home agent communication are approximately equal to *T*_*I*_. *T*_*I*_ ≈ *T*_MN-CN_ ≈ *T*_MN-HA_ ≈ *T*_HA-CN_^  ^.  

Thus, the latency of the *Binding Update* for the two protocols in terms of Internet time is

(9) LRR-RO=5TI2,LTOTP-RO=3TI2.

To update the binding in case of binding lifetime expiry, the number of messages in the improved TOTP-RO protocol is less than the normal RR-RO procedure. Consider a mobile node that moved from one network to another and performed the Route Optimization procedure. Let the binding lifetime be *t* seconds and the mobile node does not move again in a given time interval *T*. The number of RR-RO message can be approximated as discussed by [20]:

(10) NRR-RO=4∗Tt.

Similarly, the number of TOTP-RO messages can be approximated as

(11) NTOTP-RO=4∗(Tt−x),

where *x* is the number of times mobile node found the traffic for the particular binding entry for which binding lifetime has expired.

Assuming that one-fourth of the times when binding lifetime was expired, the mobile node has not found any traffic for that particular binding entry; hence, *x* = 3/4∗*T*/*t*.

**Figure 14:**
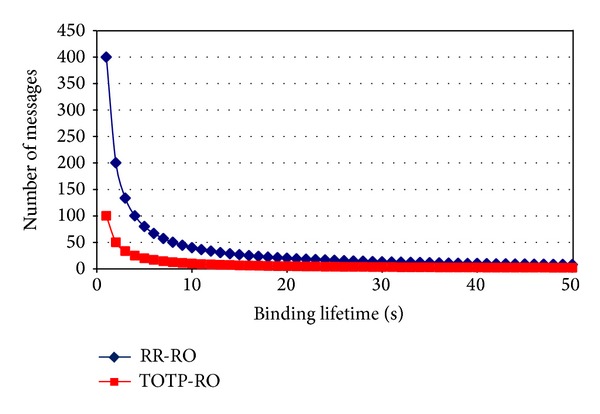
Number of messages exchanged due to binding lifetime expiry.

Also assuming that the mobile node does not move again in a given time interval *T* = 100 sec, the number of messages with varying binding lifetime for the two protocols is shown in Figure 14.

Figure 14 shows that around 75% number of messages has been reduced with the proposed TOTP-RO as compared to the RR-RO, due to not exchanging binding messages each time the binding lifetime expires.

**Figure 15:**
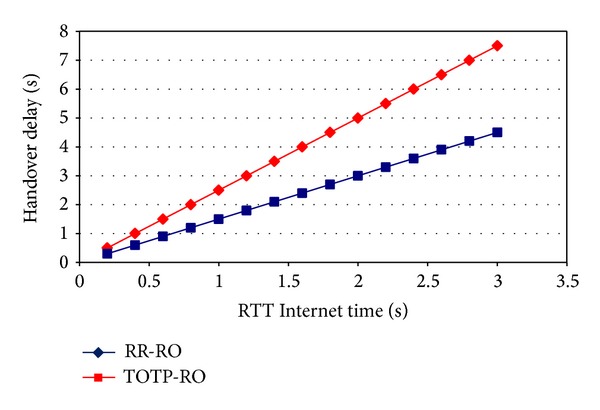
Handover delay comparison as a function of Internet time.

The Handover delay as a variant of RTT Internet time is shown in Figure 15. As the RTT value for the Internet time increases, the handover delay is also increased. For a small value of RTT Internet time, the delay caused by the RR-RO is not too high as compared to the delay caused by the TOTP-RO, but as the RTT Internet time value increases, the difference between the handover delays of two protocols is becoming high.

The comparison among RR-RO, RR-IBEP, HCBU, and TOTP-RO for the message exchange is shown in Table 2. All the protocols except TOTP-RO involve heavy message exchange for the authentication of the mobile node and the update of the binding entry at the correspondent node.

Keeping in view the number of heavy message exchange by different Route Optimization protocols, the mobile node's authentication overhead ratio induced by the mobility routing can be approximated as

(12) Overhead_Ratio=No_of_Auth_MessagesTotal_No_of_RO_Messages.

Table 3 shows the comparison of protocols. The authentication overhead for TOTP-RO is reduced to zero, as no additional signalling is done for the authentication of mobile node. Similarly, the latency (Internet time) of the TOTP-RO is also minimum as compared to other protocols.

## 6. Conclusion and Future Research Directions

Due to the problems of longer service disruption and high overhead in Mobile IPv6, this paper proposed a new solution, TOTP-RO, for the Mobile IPv6 Route Optimization. In the proposed solution, correspondent node compatibility test was performed in addition to the computation of the shared secret Token and authentication through time-based one-time password technique. The correspondent node compatibility test helped to reduce the handover signalling and delay when it was found that correspondent node does not have an active implementation of Mobile IPv6. Mobile node's authentication was achieved through a shared secret Token and time-based one-time password that resulted in direct authentication of mobile node and decreasing the overall service disruption for real-time applications. Simulations were performed in network simulator 2 (NS-2) and an analytical analysis was also made. Results showed that the proposed enhancement has significantly improved the performance with respect to handover delay, packet loss, and signalling overhead. The authentication overhead with the TOTP-RO has been reduced to 0% as compared to the existing solutions where it was around 75%. The time-based authentication will also help other mobility management protocols to protect against certain security attacks and research community can use this optimized solution for other variants of Mobile IPv6 as well to improve their performance with respect to service disruption delay and overhead in the network.

## Figures and Tables

**Table 1:** Comparison of Route Optimization protocols for security features.

Feature	RR-RO	RR-IBE	HCBU	TOTP-RO
Authentication	Weak	Strong	Strong	Strong
Time based authorization	No	No	No	Yes
Data confidentiality	No	Yes	No	No
Data integrity	No	Yes	No	No
Non-repudiation	No	Yes	Yes	Yes
Send false binding	Yes	No	No	No
MITM attack prevention	No	Yes	Yes	Yes
Amplification attack reduction	No	Yes	No	Yes
Replay attack prevention	No	Yes	No	Yes
Denial of Service prevention	No	No	Yes	Yes
Redirection attack prevention	No	No	Yes	Yes

**Table 2:** A number of messages comparison for different Route Optimization protocols.

Original RR-RO protocol:	
Packet 1: HoTI from mobile node to home agent.	
Packet 2: HoTI from home agent to correspondent node.	
Packet 3: HoT from correspondent node to home agent.	
Packet 4: HoT From home agent to mobile node.	
Packet 5: CoTI from mobile node to correspondent node.	
Packet 6: CoT from correspondent node to mobile node.	
Packet 7: BU from mobile node to correspondent node.	
Packet 8: BA from correspondent node to mobile node.	
RR-IBE protocol:	
Packet 1: P1_HoTI from mobile node to home agent.	
Packet 2: P1'_HoTI from home agent to correspondent node.	
Packet 3: P2_CoTI from mobile node to correspondent node directly.	
Packet 4: P3 from correspondent node to private key generator.	
Packet 5: P4 from private key generator to correspondent node.	
Packet 6: P5 from correspondent node to mobile node.	
Packet 7: P6 (BU) from mobile node to correspondent node.	
Packet 8: P7 (BA) from correspondent node to mobile node.	
HCBU protocol:	
Packet 1: BU_Req from mobile node to home agent.	
Packet 2: EXCH0 from the home agent to the correspondent node.	
Packet 3: EXCH1 from correspondent node to home agent.	
Packet 4: CoA Reg from mobile node to home agent.	
Packet 5: BU_Req from home agent to correspondent node.	
Packet 6: BU_Rep from home agent to mobile node.	
Packet 7: BU from mobile node to correspondent node directly.	
Packet 8: P7 (BA) from correspondent node to mobile node.	
TOTP-RO protocol:	
Packet 1: MBU from mobile node to correspondent node.	
Packet 2: BA from correspondent node to mobile node.	

**Table 3:** Comparison of Route Optimization protocols.

Protocol	Total number of messages for RO	Number of messages for authentication	Authentication overhead (%)	PKI dependence	Latency (Internet time)
RR-RO	8	6	75%	No	5/2∗*T*_*I*_
TOTP-RO	2	2	0%	No	3/2∗*T*_*I*_
RR-IBE	8	6	75%	Yes	5/2∗*T*_*I*_
HCBU	7	4	57%	No	5/2∗*T*_*I*_
